# Improving Symptoms of Peripheral Artery Disease With Hirudotherapy

**DOI:** 10.7759/cureus.16270

**Published:** 2021-07-08

**Authors:** Omar Afify, Samaa Alkhouri, Nehman Lauder

**Affiliations:** 1 Family Medicine, Wayne State University School of Medicine, Detroit, USA

**Keywords:** outpatient family medicine, peripheral arterial diseases, critical limb ischemia, leech therapy, self medication

## Abstract

Peripheral artery disease (PAD) is a significant cause of morbidity and mortality in the world. Critical limb ischemia is a complication of PAD that leads to severe pain at rest, numbness, and absent or diminished pulses in the legs or feet. Revascularization with surgery or endovascular intervention is required to re-establish blood flow to the affected areas. Failure to respond to medical and/or surgical treatment can lead to amputations. The decision to amputate one’s limb can be very challenging. Here, we report a patient with critical limb ischemia who refused a below-the-knee amputation and self-treated with medicinal leech therapy, or hirudotherapy. His symptoms including his pain, burning, and numbness improved significantly following six months of therapy.

## Introduction

Peripheral artery disease (PAD) is a disorder of widespread atherosclerosis often affecting the aorta, iliac and lower-extremity arteries that can be asymptomatic or present with signs of poor circulation [1]. Patients with PAD are often identified in an outpatient setting and require immediate intervention as PAD is associated with an increased risk of myocardial infarction and cardiovascular death [2]. Despite a prevalence of more than 200 million worldwide, PAD is still underdiagnosed and undertreated [3]. PAD can progress to chronic limb ischemia (CLI), which is associated with significant morbidity and mortality. Treatment of CLI includes ischemic pain control and revascularization with surgery, endovascular therapy, or a combination of both interventions. Wound care and reducing cardiovascular risk factors such as smoking cessation are imperative to prevent amputation [4]. Nevertheless, amputation occurs in 20% to 30% of patients with CLI [5].

While hirudotherapy or medicinal leech therapy has not been incorporated into the international guidelines of managing CLI, hirudotherapy has been shown to improve venous congestion and chronic wound healing [4-6]. The secretions of leeches have been shown to have thrombolytic, anticoagulant, and pain-relieving effects [7]. Cases have reported the role of hirudotherapy in managing venous leg ulcers, diabetic foot ulcers, and Buerger’s disease [6].

## Case presentation

**Figure 1:**
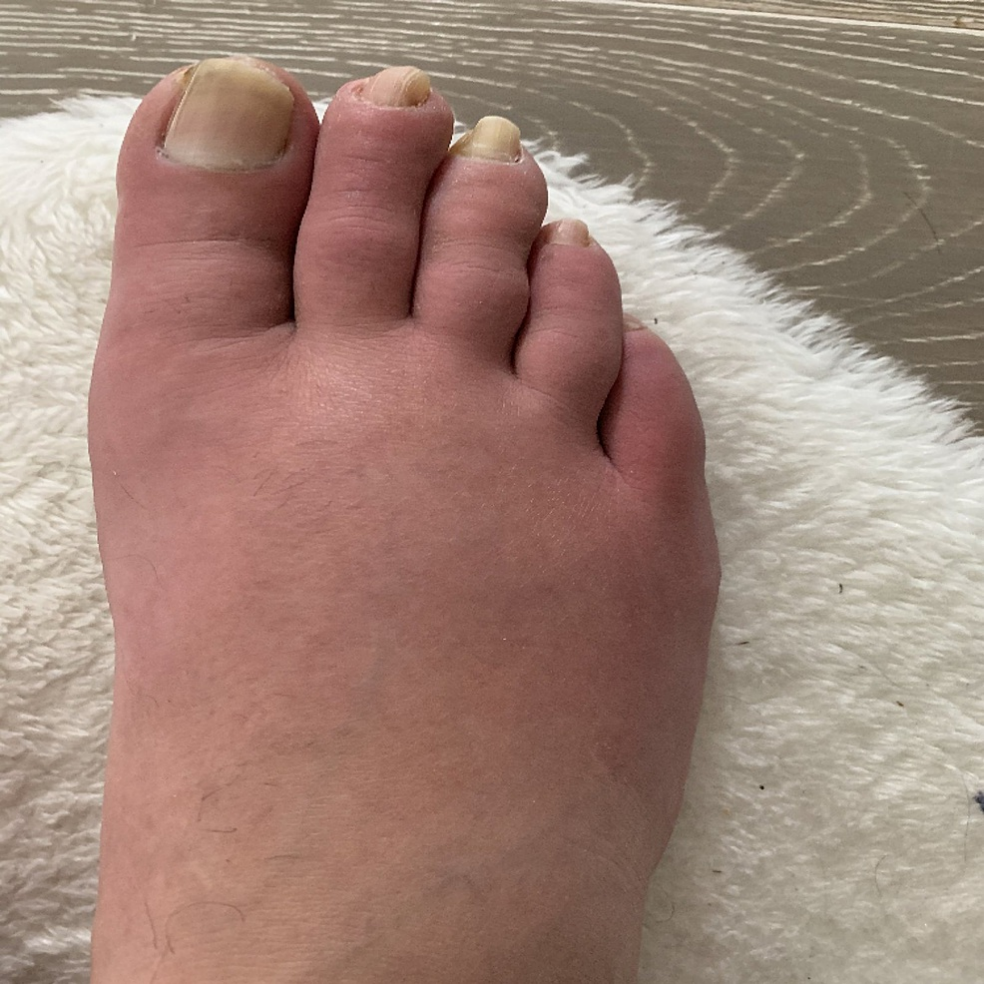
Patient presents with ischemic foot pain, redness and swelling

A 50-year-old male with a past medical history of nephrolithiasis and gout and a history of significant cigarette smoking presented to his family medicine physician with six months of progressively worsening right foot pain with associated redness and swelling (Figure 1). The patient was also experiencing burning, numbness, and tingling in his right lower extremity. After ruling out more benign causes, his family physician directed him to the local hospital for a workup of more insidious etiologies.

In the emergency department, the patient had an ankle-brachial index of 1.13 which was falsely elevated secondary to hardening of the arteries at the ankle. Moreover, a lower extremity arterial study revealed a digit pressure of 0 mmHg, suggesting critical pedal digit occlusive disease. The patient underwent a right lower extremity angiogram that showed significant chronic occlusion of the anterior tibial artery and attempts to recanalize were unsuccessful (Video 1). Vessels below the distal third of the leg were not present with the exception of a few collaterals to the foot. Since there was no flow to the foot and the occlusion was present for at least six months, vascular surgery concluded that this patient would not be amenable to endovascular nor open surgical interventions.

**Video 1:** Lower Extremity Angiogram

Following an unsuccessful revascularization, therapy shifted to hyperbaric oxygen and medical management with aspirin, atorvastatin, cilostazol, and gabapentin. The patient was also counseled about the consequences of smoking and received nicotine patches to assist with cessation. Other cardiovascular risk factors were not present. The patient was not diabetic and had a hemoglobin A1C of 5.9. Moreover, he had no history of hypertension and was normotensive throughout his admission. After failing to respond to this treatment, the patient was told that he would need a below-the-knee amputation. The patient refused and decided to explore other options including seeking a second opinion and researching online supplements.

**Figure 2:**
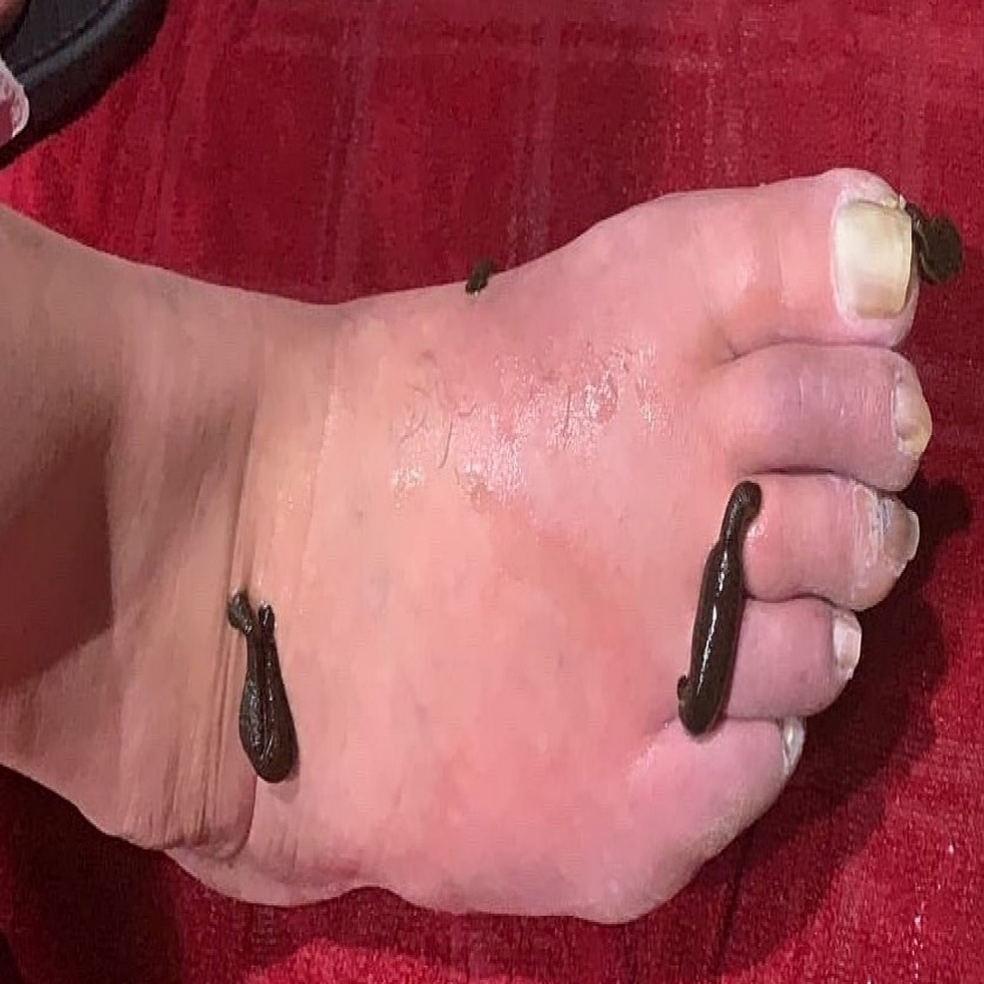
Patient places leeches on foot

After exhausting other options and treatments, the patient heard from a friend about leech therapy. Hoping to avoid amputation, the patient decided to self-treat by purchasing leeches online and placing four leeches on his foot twice daily until satiated for five days each week (Figure 2).

**Figure 3:**
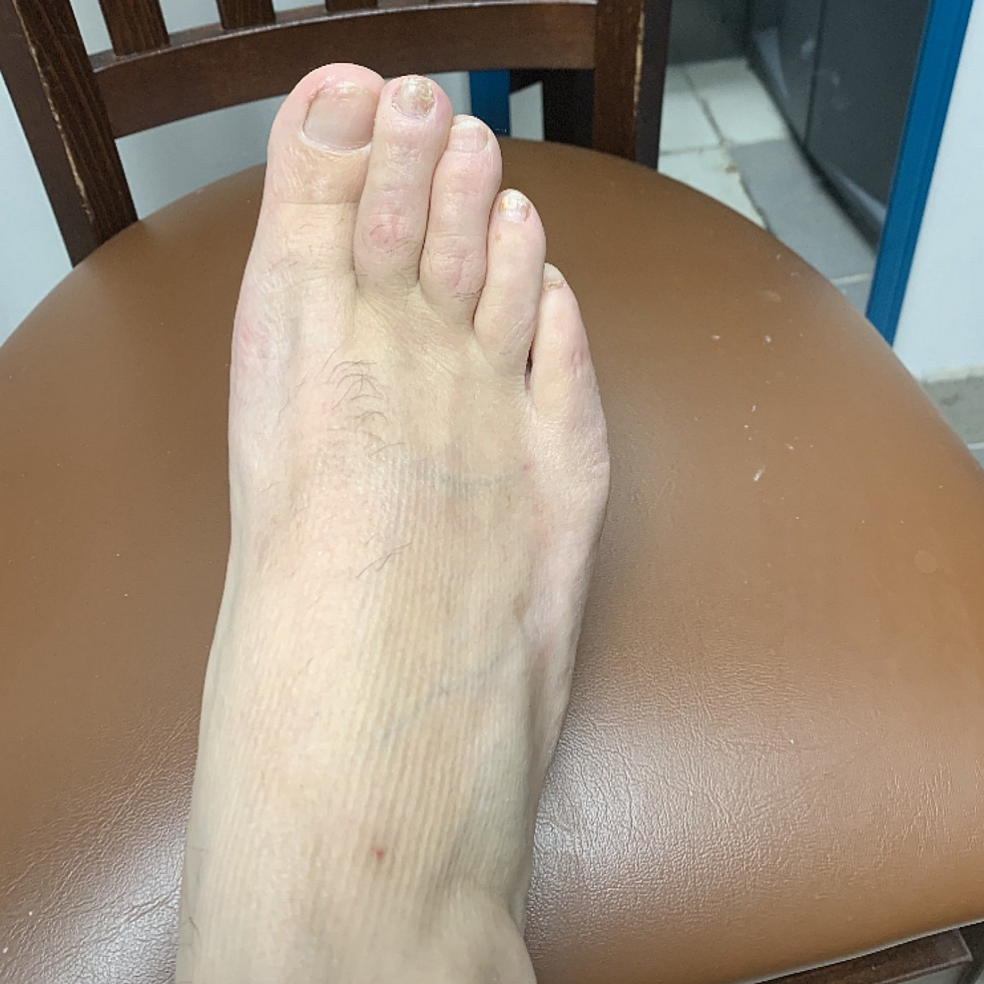
Patient's foot after six months of leech therapy

After six months, the patient presented to his family physician with improved foot pain with no numbness, burning nor tingling. The patient was not taking any medications or supplements other than completing his weekly leech therapy. On exam, the patient’s right foot was warm, dry with palpable pulses, and no cyanosis, erythema, or edema was present. The patient’s foot was remarkable for skin scars where the patient had placed the leeches (Figure 3)

## Discussion

PAD, a consequence of atherosclerosis, is associated with significant morbidity and mortality in the world and affects millions of Americans. Studies have demonstrated that PAD increases the risk of all-cause mortality by two-fold compared to those with no PAD. Despite this, PAD remains underdiagnosed, leaving many individuals unaware and, therefore, not receiving secondary prevention therapies [8]. About 10% of patients with PAD have CLI, a serious complication defined by tissue damage and/or ischemic pain at rest. Risk factors such as smoking and diabetes increase the likelihood of progression to CLI by three- and four-fold, respectively [9]. Treatment requires interdisciplinary care in order to control symptoms, manage wounds, and revascularize. Revascularization with surgical bypass or endovascular interventions is the mainstay of treatment [9]. In 20-30% of patients with CLI, amputation is required due to failure of medical treatment and revascularization [5]. Patients can refuse amputation for many reasons and may elect to self-treat including with hirudotherapy, as was the case with our patient.

Hirudotherapy or treatment with leeches is an ancient technique that has been largely ignored by modern medicine until recently. Our case report presents a 50-year-old male whose ischemic pain secondary to peripheral artery disease improved after six months of leech therapy. Many case reports have documented the effectiveness of leeches in treating a variety of diseases including inflammatory conditions, reconstructive surgeries, osteoarthritis, deep vein thrombosis, and postthrombotic syndrome [10]. The positive outcomes are presumed to be from leech secretions, in which more than 20 bioactive molecules have been identified [10].

One of the most well-studied molecules in these secretions is hirudin, a polypeptide that inhibits thrombin, a key component of the coagulation cascade. Compared to heparin, hirudin does not require antithrombin III to inactivate thrombin and is not neutralized by platelet factor 4 [7]. However, animal studies have demonstrated increased resistance following repeat exposures to hirudin. Nonetheless, bivalirudin and lepirudin have been derived from hirudin and tested as an anticoagulant for acute coronary syndromes and coronary interventions [7]. In addition to anticoagulation, leech secretions assist with tissue repair through extracellular matrix degradation due to hyaluronidase and collagenase and platelet inhibition, following saratin, calin and apyrase release [7,10]. Moreover, blood flow increases as a leech bite contains acetylcholine and histamine-like molecules. Finally, molecules such as antistasin, leech-derived tryptase inhibitor, complement C1 inhibitor and eglin in leech secretions have been shown to have anti-inflammatory properties and analgesic effects [7,10].

Due to the potential therapeutic effects, studies from Asian and Middle-Eastern regions have employed hirudotherapy to delay or avoid amputation. In one case report, a 74-year-old female with an infected diabetic foot ulcer failed medical management and was recommended to have an amputation [11]. The patient decided to undergo hirudotherapy and was completely healed in a month with no recurrence after two months of observation. Moreover, in a case series by the same authors, ten patients with second- and third-degree foot diabetic ulcers were cured and avoided amputation [12]. While these results are encouraging, the use of leech therapy is currently not recommended in the management of critical limb ischemia in the United States [13]. Other than the data provided in case reports, a review of the literature shows a paucity of information on the role of hirudotherapy in managing PAD and CLI. Randomized controlled trials (RCTs) and systematic reviews are warranted to assess and validate the effectiveness of this intervention presented in case reports.

Although these findings are promising, hirudotherapy can have serious complications with an overall rate of 21.8% [14]. The most common complications are infection and prolonged bleeding. Leeches serve as vectors for the *Aeromonas* species of bacteria, which can manifest in a variety of ways, most commonly as a gastrointestinal infection. Because of this risk, antibiotic prophylaxis is recommended when using leeches [14]. Furthermore, excessive bleeding from leech therapy can lead to significant anemia. A systematic review by Whitaker et al. reported that 49.75% of patients undergoing hirudotherapy required blood transfusions [15]. Health care providers should be aware of these risks as early medical intervention can reduce morbidity and mortality.

## Conclusions

Our case reports a 50-year-old male who presented to his family medicine physician with critical limb ischemia that required acute intervention. The patient deferred an amputation and elected to self-treat with hirudotherapy. While not recommended, the patient's symptoms, nevertheless, improved and his pain was no longer present. The literature on hirudotherapy is limited to case reports and warrants further investigation with RCTs and systematic reviews to evaluate and validate its potential benefits. Healthcare professionals should investigate hirudotherapy as a potential adjunctive therapy for limb ischemia secondary to peripheral artery disease. 

## References

[1] Olin JW, White CJ, Armstrong EJ, Kadian-Dodov D, Hiatt WR (2016). Peripheral artery disease: evolving role of exercise, medical therapy, and endovascular options. J Am Coll Cardiol.

[2] Selvin E, Erlinger TP (2004). Prevalence of and risk factors for peripheral arterial disease in the United States: results from the National Health and Nutrition Examination Survey, 1999-2000. Circulation.

[3] Shu J, Santulli G (2018). Update on peripheral artery disease: epidemiology and evidence-based facts. Atherosclerosis.

[4] Slovut DP, Sullivan TM (2008). Critical limb ischemia: medical and surgical management. Vasc Med.

[5] Woelk CJ (2012). Management of critical limb ischemia. Can Fam Physician.

[6] Koeppen D, Aurich M, Pasalar M, Rampp T (2020). Medicinal leech therapy in venous congestion and various ulcer forms: perspectives of Western, Persian and Indian medicine. J Tradit Complement Med.

[7] Whitaker IS, Cheung CK, Chahal CA, Karoo RO, Gulati A, Foo IT (2005). By what mechanism do leeches help to salvage ischaemic tissues? A review. Br J Oral Maxillofac Surg.

[8] Pande RL, Perlstein TS, Beckman JA, Creager MA (2011). Secondary prevention and mortality in peripheral artery disease: National Health and Nutrition Examination Study, 1999 to 2004. Circulation.

[9] Levin SR, Arinze N, Siracuse JJ (2020). Lower extremity critical limb ischemia: a review of clinical features and management. Trends Cardiovasc Med.

[10] Sig AK, Guney M, Uskudar Guclu A, Ozmen E (2017). Medicinal leech therapy-an overall perspective. Integr Med Res.

[11] Hajtalebi H., Iurigh H.K., Hajtalebi H.R Treatment of diabetic foot ulcer in a 74-year-old female patient based on Iranian traditional medicine in Bojnurd. Asian J Clin Case Rep Trad Alter Med.

[12] Hajtalebi H., Iurigh H.K., Hajtalebi H.R (2017). Investigating the treatment status of 10 patients with second and third degree diabetic foot ulcers based on the therapeutic protocols of Iranian traditional medicine in Bojnurd from 2015-2016. Asian J Clin Case Rep Trad Alter Med.

[13] Kinlay S (2016). Management of critical limb ischemia. Circ Cardiovasc Interv.

[14] Hackenberger PN, Janis JE (2019). A comprehensive review of medicinal leeches in plastic and reconstructive surgery. Plast Reconstr Surg Glob Open.

[15] Whitaker IS, Oboumarzouk O, Rozen WM, Naderi N, Balasubramanian SP, Azzopardi EA, Kon M (2012). The efficacy of medicinal leeches in plastic and reconstructive surgery: a systematic review of 277 reported clinical cases. Microsurgery.

